# A Novel Digital Technique for Measuring the Accuracy of an Indirect Bonding Technique Using Fixed Buccal Multibracket Appliances

**DOI:** 10.3390/jpm11090932

**Published:** 2021-09-18

**Authors:** Ignacio Faus-Matoses, Clara Guinot Barona, Álvaro Zubizarreta-Macho, Vanessa Paredes-Gallardo, Vicente Faus-Matoses

**Affiliations:** 1 Department of Stomatology, Faculty of Medicine and Dentistry, University of Valencia, 46010 Valencia, Spain; ignacio.faus@uv.es (I.F.-M.); vanessa.paredes@uv.es (V.P.-G.); vicente.faus@uv.es (V.F.-M.); 2 Department of Orthodontics, Faculty of Medicine and Health Sciences, Catholic University of Valencia, 46001 Valencia, Spain; clara.guinot@ucv.es; 3 Department of Endodontics, Faculty of Health Sciences, Alfonso X El Sabio University, 28691 Madrid, Spain; 4 Department of Surgery, Faculty of Medicine and Dentistry, University of Salamanca, 37008 Salamanca, Spain

**Keywords:** orthodontics, digital, morphometry, accuracy, indirect bonding

## Abstract

The aim of this study was to analyze the accuracy and predictability of the indirect bonding technique of fixed buccal multibracket appliances using a customized iterative closest point algorithm. Materials and Methods: A total of 340 fixed buccal multibracket appliances were virtually planned and bonded on 34 experimental anatomically based acrylic resin models by using orthodontic templates designed and manufactured to indirectly bond the fixed buccal multibracket appliances. Afterwards, the models were submitted to a three-dimensional impression technique by an intraoral scanner, and the standard tessellation language digital files from the virtual planning and the digital impression were aligned, segmented, and realigned using morphometric software. Linear positioning deviations (mm) of the fixed buccal multibracket appliances were quantified at mesio-distal, bucco-lingual/palatal, and gingival/occlusal (vertical) planes, and angular deviations (°) were also recorded by analyzing the torque, tip, and rotation using a customized iterative closest point algorithm, the script for which allowed for an accuracy measurement procedure by comparing the tessellation network positioning of both standard tessellation language digital files. Results: The mean mesio-distal deviation was −0.065 ± 0.081 mm, the mean bucco-lingual/palatal deviation was 0.129 ± 0.06 m, the mean vertical deviation was −0.094 ± 0.147 mm, the mean torque deviation was −0.826 ± 1.721°, the mean tip deviation was −0.271 ± 0.920°, and the mean rotation deviation was −0.707 ± 0.648°. Conclusion: The indirect bonding technique provides accurate and predictable positioning of fixed buccal multibracket appliances.

## 1. Introduction

It was reported that the outcome of orthodontic treatment is directly related to the correct positioning of fixed multibracket appliances on the enamel surface of the clinical crown of teeth [1]. Furthermore, the accuracy of the fixed multibracket appliance placement has been considered a highly relevant factor for the predictability of tooth movement since the edgewise archwire was developed in 1928 by Angle [2]. Consequently, Nawrocka et al. [3], Duarte et al. [4], and Guenther and Larson [5] highlighted the accuracy of the fixed multibracket appliance placement at the beginning of the orthodontic treatment and reported its influence on the outcome of the tooth positioning at the end of the orthodontic treatment. Hence, the accuracy of direct and indirect bonding procedures on fixed multibracket appliance placement and, therefore, on the predictability of tooth movement, was analyzed [6]. The direct bonding method consists of a one-stage procedure, involving placing the fixed multibracket appliance directly on the enamel surface [5]; however, the digital indirect bonding procedure requires a digital planning approach of the fixed multibracket appliance placement, using specific orthodontic planning software before transferring the information of the bracket positioning using a three-dimensional printed template [4,5]. Moreover, the indirect bonding procedure has resulted in significantly (*p* ≤ 0.05) lower deviation values (−0.20 ± 0.8 mm, −0.05 ± 0.10 mm, and 0.02 ± 0.05° in the vertical, horizontal, and angular planes, respectively) compared to conventional direct bonding procedures (−0.27 ± 0.46 mm, −0.11 ± 0.30 mm, and 0.08 ± 0.14° in the vertical, horizontal, and angular planes, respectively) [6]. Additionally, it requires fewer fixed multibracket appliance placement corrections during the orthodontic treatment, reducing working time and making the experience more comfortable for the patient [1,7,8,9]. Furthermore, the adjustment of the three-dimensional printed template, the doctor experience, the three-dimensional printed template material, and the isolation conditions can affect the accuracy of the three-dimensional printed template, and finally, the outcome of the fixed multibracket appliance placement [10].

Other authors previously analyzed the movements performed by orthodontic appliances by cone-beam computed tomography (CBCT) scans [11]. Moreover, electromyographic data and balance tests were also used to assess the treatment outcome [12]. However, the development of digital technology has led to the generation of accurate Standard Tessellation Language (STL) digital files, which have been used to analyze the ideal position of the teeth in a virtual setup and to determine, virtually, the best location of the fixed multibracket appliance on the teeth [13]. Moreover, it allows for the design and manufacturing of a three-dimensional printed template to perform indirect bonding of the fixed multibracket appliances, a technique that has emerged as a new era in daily practice [13]. The main advantages of the indirect bonding procedure of fixed multibracket appliances have been clearly described in the literature; however, information about bracket transfer accuracy is limited, and therefore, it is necessary to develop studies to analyze the accuracy of the indirect bonding procedure of fixed multibracket appliances.

The aim of this work was to analyze the accuracy and predictability of the indirect bonding technique of fixed buccal multibracket appliances by a novel digital measurement technique, with a null hypothesis (H_0_) stating that the indirect bonding technique does not provide accurate and predictable positioning of fixed buccal multibracket appliances.

## 2. Materials and Methods

### 2.1. Study Design

A total of 340 fixed buccal brackets (Clarity™ Advanced, 3M Corp.) were bonded on 34 experimental anatomically based acrylic resin models (17 maxilla and 17 mandibles) with 14 clinical crowns each, selected for this study at the Department of Stomatology at the University of Valencia (Valencia, Spain), between November 2020 and February 2021.

### 2.2. Experimental Procedure

**Figure 1:**
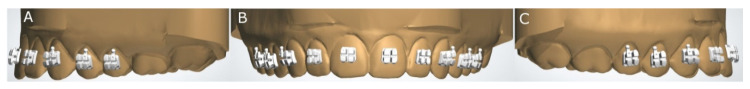
(**A**) Lateral left, (**B**) frontal, and (**C**) lateral right view of the fixed buccal multibracket appliances digitally planned on the experimental anatomically based models.

The maxillary and mandibular experimental anatomically based acrylic resin models were designed by 2D/3D computer-aided design/computer-aided engineering (CAD/CAE) (Midas FX+^®^, Brunleys, MK, UK) and manufactured using a three-dimensional impression technique (ProJet^®^ 6000. 3D Systems©, Rock Hill, SC, USA). Afterwards, the fixed buccal multibracket appliance (Clarity™ Advance, 3M, St. Paul, MI, USA) placement was digitally planned using orthodontic planning software (Ortho Analyzer, 3Shape, Copenhagen, Denmark) (Figure 1A–C).

**Figure 2:**
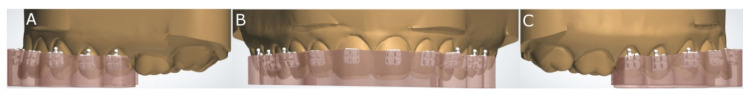
(**A**) Lateral left, (**B**) frontal, and (**C**) lateral right view of the orthodontic template digitally planned on the experimental anatomically based models.

Then, digital orthodontic templates were also designed using orthodontic planning software (OrthoAnalyzer, 3shape, Copenhagen, Denmark) (Figure 2A–C).

**Figure 3:**
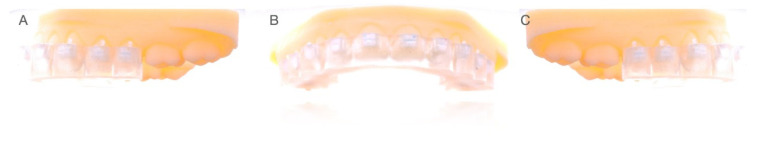
(**A**) Lateral left, (**B**) frontal, and (**C**) lateral right view of the fixed buccal multibracket appliances with the bonding trays on the anatomically based acrylic resin models.

Subsequently, the orthodontic templates were manufactured using a three-dimensional impression technique (ProJet^®^ 6000. 3D Systems©, Rock Hill, SC, USA) in an acrylic resin (Ortho IBT, NextDent, Soesterberg, The Netherlands) that was polymerized for 10 min, following the manufacturer’s recommendations (Figure 3A–C).

Finally, the fixed buccal multibracket appliances (Clarity™ Advanced, 3M Corp) were bonded on both the maxilla (from tooth 1.5 to 2.5) and mandible (from tooth 3.5 to 4.5) experimental models in the center of the buccal surface of the clinical crown with the photo-polymerized composite resin cement incorporated into the mesh of the brackets (APC™ FlashFree Adhesive, 3M Corp.), and without etching, resin adhesive application or additional photo-polymerized composite resin cement application. Then, all experimental anatomically based acrylic resin models were submitted to a digital impression using an intraoral scan (Trios, 3Shape, Copenhagen, Denmark) by means of 3D in-motion video imaging technology to obtain an accurate standard tessellation language (STL) digital file. The image capturing procedure was performed, following the manufacturer’s recommendations, by scanning the occlusal plane first, followed by the buccal and palatal surfaces.

### 2.3. Alignment Procedure

**Figure 4:**
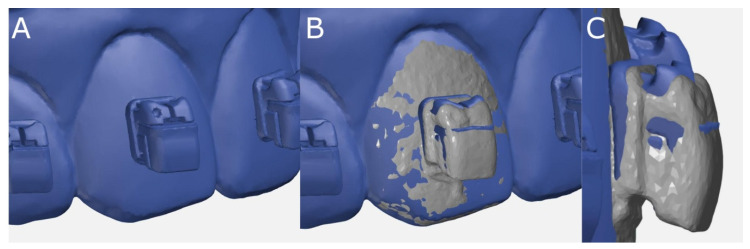
(**A**) The STL digital file of the fixed buccal multibracket appliances digitally planned on the experimental anatomically based models. (**B**) Front and (**C**) right lateral views of the alignment procedure of the STL digital file from the virtually planned position of the digitalized fixed buccal multibracket appliances and the STL digital file from the digital impression of the fixed buccal multibracket appliances bonded to the experimental anatomically based models.

Afterwards, the STL digital files were imported to a reverse engineering morphometric software (3D Geomagic Capture Wrap, 3D Systems©, Rock Hill, SC, USA) and the teeth were individually segmented to allow for the accuracy alignment procedure between the STL digital files from the virtually planned position of the digitalized fixed buccal multibracket appliances (Clarity Advanced, 3M Corp.) and the STL digital files of the fixed buccal multibracket appliances (Clarity Advanced + APC Flash-Free, 3M Corp) bonded to the experimental anatomically based acrylic resin models, according to the recommendations of Zubizarreta-Macho et al. [14]. The alignment procedure was performed using the palatal surfaces of the anterior teeth and the occlusal and palatal surfaces of the posterior teeth with the best-fit algorithm (Figure 4).

### 2.4. Measurement Procedure

**Figure 5:**
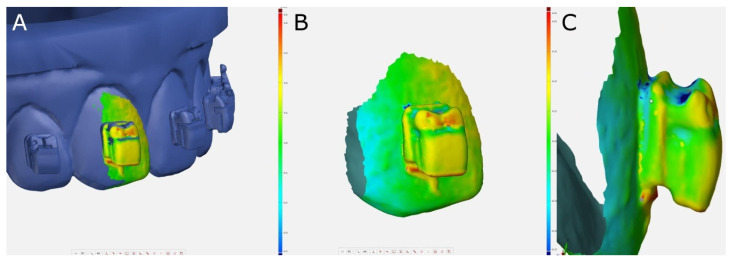
(**A**) Measurement procedure. (**B**) Frontal and (**C**) right lateral views of the segmented tooth 1.1. Warm colors represent a volume increase, cold colors represent a volume decrease, and green represents an accurate alignment.

After the alignment procedure was performed, the following variables were measured: linear deviations (mm)—mesio-distal, bucco-lingual/palatal, and gingival/occlusal (vertical); angular deviations (°)—torque, tip, and rotation (Figure 5).

Linear deviations in the mesial, buccal, or incisal/occlusal directions were recorded with positive values, and linear deviations in the distal, lingual/palatal, or gingival directions were recorded with negative values. Angular deviations in the mesial or incisal/occlusal directions were recorded with positive values, and angular deviations in the distal or gingival directions were recorded with negative values. In addition, a crown-tip inadequacy of 2° causes a marginal ridge discrepancy of 0.5 mm [3,14]. Therefore, linear deviations of ≤0.5 mm and angular deviations of ≤2° were used as the criteria.

**Figure 6:**
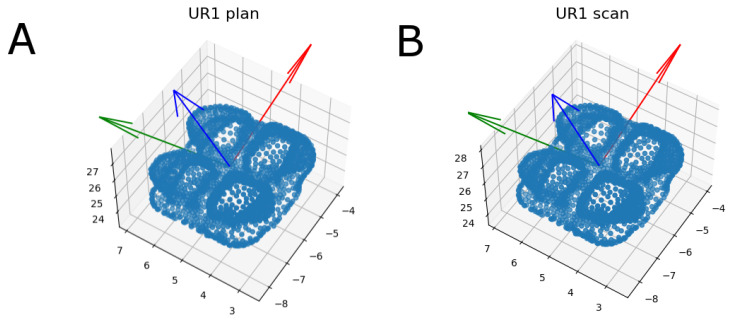
(**A**) Planned and (**B**) scanned bracket cloud of points representing the three-dimensional position of bracket 3.2. Blue points represent the vertices of the tessellations of equilateral triangles.

Moreover, the measurement procedure was performed using an iterative closest point (ICP) algorithm, the script of which allowed for an accurate measurement procedure by comparing the tessellation network positioning of both STL digital files (Figure 6).

### 2.5. Statistical Analysis

The statistical analysis of the measurement variables was carried out using SAS 9.4 (SAS Institute Inc., Cary, NC, USA). Descriptive statistics were expressed as the mean and standard deviation (SD) values for the quantitative variables.

## 3. Results

The mean and SD values for the linear deviation measurements (mm) of the STL digital files are displayed in Table 1.

**Figure 7:**
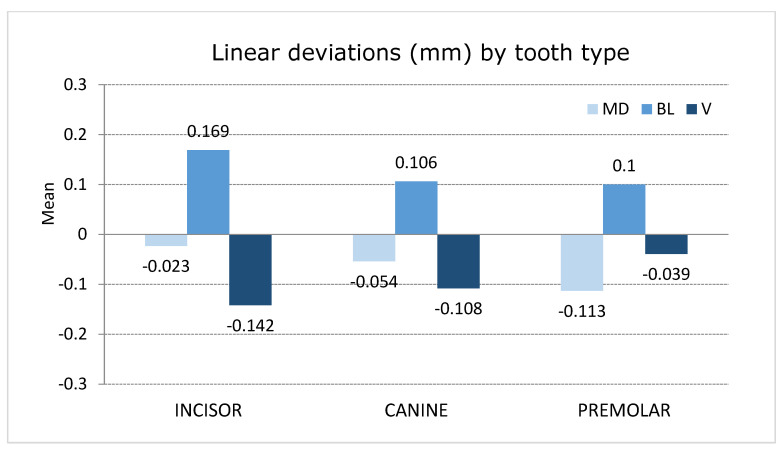
Plot of the mean values of the mesio-distal, bucco-lingual/palatal, and vertical deviations of incisors, canines, and premolars.

Premolars showed higher mesio-distal deviation values (−0.113 ± 0.084 mm) compared to canines (−0.054 ± 0.059 mm) and incisors (−0.23 ± 0.059 mm) (Figure 7). Notably, the upper right second premolar showed the highest mesio-distal deviation (−0.230 ± 0.055 mm). However, the prevalence of mesio-distal deviations within 0.5 mm of tolerance was 100%, regardless of the tooth type and maxilla. Moreover, most of the mesio-distal deviations (79.1%) were recorded as negative values.

Incisors showed higher bucco-lingual deviation values (0.169 ± 0.0.64 mm) than canines (0.106 ± 0.052 mm) and premolars (0.100 ± 0.054 mm) (Figure 7). Notably, the upper right central incisor showed the highest bucco-lingual deviation (0.261 ± 0.043 mm). However, the prevalence of bucco-lingual deviations within 0.5 mm of tolerance was 100%, regardless of the tooth type and maxilla. Moreover, all of the bucco-lingual deviations (100%) were recorded as positive values.

Incisors showed higher vertical deviation values (−0,142 ± 0.159 mm) compared to canines (−0.108 ± 0.118 mm) and premolars (−0.039 ± 0.128 mm) (Figure 7). Notably, the upper left central incisor showed the highest vertical deviation (−0.224 ± 0.147 mm). However, the prevalence of vertical deviations within 0.5 mm of tolerance was 97.9%, regardless of the tooth type and maxilla. Moreover, most of the vertical deviations (83.3%) were recorded as negative values.

The mean and SD values for the angular deviation measurements (°) of the STL digital files are displayed in Table 2.

**Figure 8:**
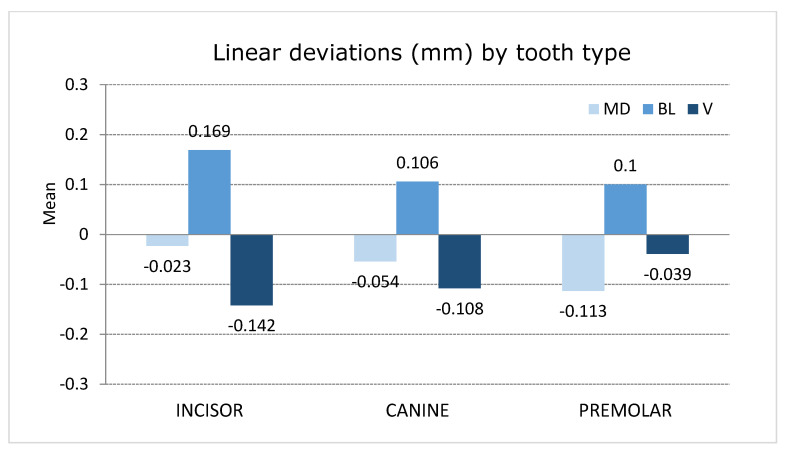
Plot of the mean values of the torque, tip, and rotation deviations of incisors, canines, and premolars.

Incisors showed higher torque values (−1.747 ± 1.538°) compared to canines (−0.889 ± 1.194°) and premolars (0.140 ± 1.604°) (Figure 8). Notably, the upper right central incisor showed the highest torque deviation (3.680 ± 0.748°). However, the prevalence of torque deviations within 2° of tolerance was 75.2% since the incisors presented a prevalence of torque deviations within 2° of tolerance of 58.5%. Moreover, most of the mesio-distal deviations (72.5%) were recorded as negative values.

Incisors showed higher tip deviation values (−0.796 ± 0.805°) compared to canines (−0.434 ± 0.590°) and premolars (0.343 ± 0.795°) (Figure 8). Notably, the upper left lateral incisor showed the highest tip deviation (1.808 ± 0.636°). However, the prevalence of tip deviations within 2° of tolerance was 75.2% since the incisors presented a prevalence of tip deviations within 2° of tolerance of 96.7%. Moreover, most of the tip deviations (63.3%) were recorded as negative values.

Canines showed higher rotation deviation values (1.038 ± 0.528°) compared to incisors (0.775 ± 0.630°) and premolars (0.471 ± 0.634°) (Figure 8). Notably, the upper right canine showed a rotation deviation of 1.194 ± 0.495° (Figure 8). However, the prevalence of rotation deviations within 2° of tolerance was 97.6%. Moreover, most of the rotation deviations (87.5%) were recorded as positive values.

## 4. Discussion

The results obtained in the present study rejected the null hypothesis (H_0_) that stated that the indirect bonding technique does not provide accurate and predictable positioning of fixed buccal multibracket appliances.

Indirect bonding of fixed multibracket appliances was developed as an alternative to conventional direct bonding procedures to provide more predictable tooth movements by using less wire bending to align the teeth and requiring less bracket rebonding near the end of the orthodontic treatment [1,15,16]. However, the transference of the digital planning of the fixed multibracket appliance placement to the dental surface may lead to inaccuracies, and hence, to unpredictable tooth movements [3,10]. Therefore, it is mandatory to analyze the discrepancy between the digital plan and the clinical position of the fixed multibracket appliances. According to Armstrong et al. (2007), differences smaller than or equal to 0.5 mm would not have any clinical significance; however, they accept this finding for all teeth except the upper and lower central incisors, for which changes smaller than or equal to 0.25 mm could have clinical consequences [17]. Nevertheless, these discrepancies have to be analyzed from the indirect bonding perspective because these discrepancies would be higher if a direct bonding technique was used, as it was found by Hodge et al. [6]. Niu et al. reported that the transference of the digital planning position of the fixed multibracket appliances to the dental surface by indirect bonding procedures still pertains to and can affect the final position of the fixed multibracket appliance on the tooth [10]. Printing workflow techniques have been highlighted as one of the main factors that could influence the accuracy of the fixed multibracket appliance position [3]. In addition, operator experience [10] and the manufactured material of the orthodontic template [1,18] have also been reported to be etiological factors in the inaccuracy of fixed multibracket appliances’ bonding positions. In this study, the bonding procedures of the fixed multibracket appliances were performed by a single operator using orthodontic templates printed by the same workflow technique and manufactured material to prevent bias [10].

Grünheid et al. reported that the anterior teeth (−0.012 ± 0.009 mm in mesio-distal measures, 0.026 ± 0.013 mm in vertical measures, and 0.0103° ± 0.085, 0.061° ± 0.005, and 0.235° ± 0.010 for the torque, tip, and rotation, respectively) showed more accurate indirect bonding positions of the fixed multibracket appliances than the posterior teeth did (−0.063 ± 0.033 mm in mesio-distal measures, 0.035 ± 0.017 mm in vertical measures, and −0.268° ± 0.520, −0.213° ± 0.445, and 0.405° ± 0.335 for the torque, tip, and rotation, respectively) [19]. Süpple et al. found more displacement in the molar teeth 21, and Kim et al. reported this discrepancy in the posterior teeth in the height of the cusps [20]. However, in the present study, this result was found just in the mesio-distal measurement. These findings can be partially explained because the molars were not included in the analysis of the present study, and the previous authors included the first molars, reporting the most directionally biased ones. In addition, the bonding capacity and the pressure applied by the operator are both difficult to control on molars, raising the lack of accuracy for the posterior areas when the anterior adjustments are compared [21].

The accuracy of the indirect bonding technique of fixed buccal multibracket appliances was determined as acceptable in all the measures except for the torque in upper central incisors, upper lateral right incisors, lower left central incisor, and lower left second premolar [19]. In this study, the biggest differences in the bonding of the brackets were in the torque. This was also found in the study of Niu et al. in which they explained that the consistency of the bonding resin adhesive between the base and the tooth surface could change the final torque position [10]. Furthermore, Grünheid et al. reported that most vertical errors have a negative sign, meaning the deviation tends to be more gingival to the planned position. This fact could be explained by the flexibility of the orthodontic template, which could be depressed under pressure, leading to a more gingival position if there is not an occlusal stop to adapt the orthodontic template [21]. Castilla et al. found that the most significant differences were in the upper left central incisor and in the first upper right molar, which is in accordance with the findings obtained in this study [21]. However, Duarte et al. found statistically significant differences (*p* ≤ 0.05) in the horizontal measurements compared to the vertical and angular measurements, which may be attributable to the multi-operator variability [4]. Moreover, Grünheid et al. also used some operators with limited clinical experience in orthodontics and indirect bonding [21]. The present study was designed by a single operator with several years of clinical experience and indirect bonding procedures, so this can explain the high level of accuracy, according to De Oliveira [22]. For future studies, it would be interesting to analyze different dental adhesives in order to evaluate the accuracy of the tray with different dental cements using the same digital tray and the same operator [23].

## 5. Conclusions

In conclusion, within the limitations of this study, the results show that the indirect bonding technique provides clinically accurate and predictable positioning of fixed buccal multibracket appliances.

## Figures and Tables

**Table 1:** Descriptive statistics of the linear deviation measurements (mm) of the STL digital files.

	*n*	Mean	SD	Minimum	Maximum
Mesio-distal	335	−0.065	0.081	−0.318	0.124
Bucco-lingual	335	0.129	0.067	0.043	0.395
Vertical	335	−0.094	0.147	−0.823	0.703

SD: standard deviation.

**Table 2:** Descriptive statistics of the angular deviation measurements (°) of the STL digital files.

	*n*	Mean	SD	Minimum	Maximum
Torque	335	−0.826	1.721	−5.177	7.091
Tip	335	−0.271	0.920	−3.970	2.463
Rotation	335	−0.707	0.648	−1.600	2.226

SD: standard deviation.

## Data Availability

Data available on request due to restrictions (e.g., privacy or ethical).
